# Serum Free Thiols Are Superior to Fecal Calprotectin in Reflecting Endoscopic Disease Activity in Inflammatory Bowel Disease

**DOI:** 10.3390/antiox8090351

**Published:** 2019-09-01

**Authors:** Arno R. Bourgonje, Ruben Y. Gabriëls, Martin H. de Borst, Marian L. C. Bulthuis, Klaas Nico Faber, Harry van Goor, Gerard Dijkstra

**Affiliations:** 1 Department of Gastroenterology and Hepatology, University of Groningen, University Medical Center Groningen, 9713 GZ Groningen, The Netherlands; 2 Department of Internal Medicine, Division of Nephrology, University of Groningen, University Medical Center Groningen, 9713 GZ Groningen, The Netherlands; 3 Department of Pathology and Medical Biology, University of Groningen, University Medical Center Groningen, 9713 GZ Groningen, The Netherlands

**Keywords:** inflammatory bowel disease, oxidative stress, biomarkers, free thiols, disease activity, fecal calprotectin

## Abstract

Oxidative stress plays a pivotal role in the pathogenesis of inflammatory bowel diseases (IBD). Serum free thiols (R-SH) reliably reflect systemic oxidative stress, since they are readily oxidized by reactive species. Here, we aimed to establish concentrations of serum free thiols in IBD and assessed their discriminating capacity regarding endoscopic disease activity. Albumin-adjusted serum free thiol concentrations were measured in 78 IBD patients (31 Crohn’s disease (CD) and 47 ulcerative colitis (UC) patients) and 50 healthy controls and analyzed for associations with disease parameters and their discriminative value regarding endoscopic disease activity (*n* = 54) or fecal calprotectin (*n* = 36) in patients for which those data were available. Mean serum free thiol concentrations were significantly lower in both CD and UC as compared to healthy controls (19.4 ± 3.1 and 17.8 ± 3.4 vs. 21.1 ± 1.9 µmol/g albumin, *P* < 0.001). Free thiols highly accurately discriminated between mild and moderate-to-severe disease activity, better than fecal calprotectin (FC) levels (AUC = 0.87, *P* < 0.001 vs. AUC = 0.76, *P* < 0.05, respectively) and this was maintained after cross-validation (AUC = 0.89, *P* < 0.001). Serum free thiols are reduced in IBD as compared to healthy controls and strongly correlate with the degree of endoscopic disease activity. Quantifying systemic redox status in IBD may be a promising, minimally invasive strategy to monitor IBD disease activity.

## 1. Introduction

Inflammatory bowel diseases (IBD), such as Crohn’s disease (CD) and ulcerative colitis (UC), are chronic inflammatory intestinal disorders that are characterized by an inappropriate and ongoing immune response, driven by dysregulated gut microbiota in genetically susceptible individuals [1]. Individuals affected by IBD typically show a disease course that consists of alternating exacerbations and periods of remission [2]. It is challenging to accurately predict disease exacerbations due to their highly variable symptomatology and the inadequate availability of surrogate disease biomarkers [3]. Most importantly, many IBD patients experience chronic subclinical disease activity, which may be detrimental to patients’ health and increases the risk of disease complications and surgical interventions [4].

To determine the presence and extent of IBD disease activity, endoscopy is the gold-standard diagnostic modality [5]. However, repeated endoscopic examination is accompanied by poor patient acceptance, due to its invasive nature and the need for bowel preparation, sedation and a small risk of complications. Moreover, it is a costly and time-consuming procedure. Clinical disease indices are commonly applied to quantify patient-reported disease activity, though they fail to accurately represent mucosal inflammation [6,7]. Hence, the diagnostic and predictive performances of surrogate markers for endoscopic disease activity are increasingly being scrutinized. In this respect, fecal calprotectin (FC) is currently considered to be the best discriminating biomarker for endoscopically proven inflammatory disease activity [8,9]. Nevertheless, both its diagnostic accuracy for differentiating between different degrees of disease activity and its clinical applicability are still subject to debate [10,11,12]. As a consequence, novel surrogate markers for active mucosal inflammation are actively sought for to monitor disease activity and detect IBD exacerbations early.

Oxidative stress, characterized by an excessive production of reactive oxygen species (ROS) and decreased antioxidant availability, is believed to play a pivotal role in IBD etiology and progression [13,14]. Systemic oxidative stress is associated with decreased levels of reduced free thiols, since thiols are rapidly oxidized by circulating reactive species [15]. Serum free thiols (R-SH, sulfhydryl groups) are a robust and powerful read-out of the systemic in vivo reduction-oxidation (redox) status [16]. Blood proteins, mainly albumin, harbor the largest amount of redox-active thiol groups (approximately 75% of the total thiol pool) [17]. The remainder is accounted for by the group of low-molecular-weight (LMW) thiols, such as cysteine, glutathione and homocysteine. These LMW thiols are present at very low concentrations in the extracellular environment, and predominantly circulate complexed with albumin [16]. Typically, high extracellular concentrations of reduced free thiols are representative of a healthy redox status.

Previous studies have examined systemic free thiol levels in a variety of conditions, including cardiovascular, renal, metabolic and rheumatological diseases [18,19,20,21]. These studies show positive associations between free thiol levels and favorable disease outcomes. On the other hand, decreased free thiols have been linked to cardiovascular risk factors, such as ageing, obesity and alcohol consumption [22,23]. Earlier, we already demonstrated that CD patients had markedly reduced levels of systemic free thiols, even in clinical remission [24]. However, since that CD cohort lacked sufficient information about endoscopic disease activity, we could not fully evaluate the possible role of free thiols as biomarker for mucosal disease activity. Over the past decades, many studies have been performed to assess oxidative stress biomarkers in IBD, though few of them addressed the potential ability of such biomarkers to categorize different grades of endoscopic disease activity in IBD [25,26].

In the present study, we aimed to determine and compare systemic free thiol concentrations between CD, UC and healthy individuals and to establish associations between serum free thiol concentrations and IBD-specific disease parameters, especially those related to inflammatory disease activity. Finally, we aimed to assess the discriminative accuracy of serum free thiol levels with regard to different degrees of endoscopic disease activity in IBD and compare this to fecal calprotectin levels.

## 2. Materials and Methods 

### 2.1. Study Population

From October 2014 until December 2018, 78 IBD patients aged 18–65 years were invited to participate in the study at the IBD outpatient clinic of the University Medical Center Groningen (UMCG), Groningen, The Netherlands. All patients had an established IBD diagnosis existing for at least 1 year according to clinical, endoscopic and histopathological criteria [27]. At time of inclusion, all patients presented with at least some degree of active disease necessitating new biological therapy, and 56.4% of patients were treated with conventional IBD treatment (e.g., mesalamine, thiopurines, or any combination of these drugs).

### 2.2. Data Collection

Demographic characteristics of the participants were collected, including age, sex, body mass index (BMI, body weight divided by squared height) and smoking status. Clinical characteristics were registered for the complete IBD cohort, including the Montreal disease classification, IBD maintenance medication (mesalamine, thiopurines, or any combination of these drugs), prior use of TNF-antagonists, IBD-related surgical history (ileocecal resection for CD and subtotal colectomy for UC) and clinical disease indices (Harvey Bradshaw index (HBI) for CD and Simple Clinical Colitis Activity Index (SCCAI) for UC) [28,29]. Disease location according to the Montreal classification was registered from the most recently performed endoscopic assessment, at least within 12 months of serum analysis. CD patients having upper gastrointestinal disease or active perianal disease were not included in the study. Standard laboratory measurements were performed in all participants, including hemoglobin (Hb), C-reactive protein (CRP), white blood cell count (WBC), platelet count, albumin and creatinine (Roche Modular, Roche, Mannheim, Germany). The estimated glomerular filtration rate (eGFR) was calculated according to the Chronic Kidney Disease Epidemiology Collaboration (CKD-EPI) equation [30]. In the IBD cohort, fecal calprotectin (FC) levels were detected in a subgroup of patients (*n* = 36) using enzyme-linked immunosorbent assays (ELISA) (Bühlmann Laboratories AG, Switzerland). In another subset of IBD patients (69.2%, *n* = 54), endoscopic disease activity was assessed within 3 months prior to serum sample analysis (77.7% within 1 month). Endoscopic disease activity was graded according to the validated Simplified Endoscopic Score for Crohn’s disease (SES-CD) for CD or the Mayo endoscopic subscore for UC [31,32], based on available endoscopic images and endoscopy reports written by certified gastroenterologists from our university hospital. All included patients who underwent endoscopic investigation had at least mild disease activity. Consequently, SES-CD scores fell into one of the following three activity categories: (1) mild (SES-CD 4–10), (2) moderate (SES-CD 11–19) or (3) severe (SES-CD ≥ 20) [33]. Similarly, Mayo endoscopic subscores are covered in the three categories (1) mild (Mayo 1), (2) moderate (Mayo 2) and (3) severe (Mayo 3). Additionally, for the purpose of total (IBD) cohort analyses, CD and UC patients falling into the same endoscopic categories were merged to create a composite IBD endoscopic disease activity assessment.

### 2.3. Measurement of Serum Free Thiols

Serum samples were stored at −80°C until free thiol analysis. Sample storage at this temperature does not significantly alter free thiol stability. Samples from IBD patients and non-IBD controls were obtained according to the same protocols. Serum free thiol concentrations were measured as previously described, with few modifications [34,35]. As a first step, serum samples were diluted 4-fold using 0.1 M Tris buffer (pH 8.2). After measuring background absorption at 412 nm using the Varioskan microplate reader (Thermo Scientific, Breda, The Netherlands), together with a reference measurement at 630 nm, 20 µL 1.9 mM 5,5’-dithio-bis (2-nitrobenzoic acid) (DTNB, Ellman’s Reagent, CAS-number 69-78-3, Sigma Aldrich Corporation, St. Louis, MO, USA) in 0.1 M phosphate buffer (pH 7.0) was added to the samples. Absorbance was measured again after an incubation time of 20 min at room temperature. Final concentrations of free thiol groups were determined by parallel measurement of an l-cysteine (CAS-number 52-90-4, Fluka Biochemika, Buchs, Switzerland) calibration curve (concentration range from 15.6 µM to 1000 µM) in 0.1 M Tris/10 mM EDTA (pH 8.2). Both intra- and interday coefficients of variation (CV) of the free thiol measurements were <10%. Finally, serum free thiol concentrations were adjusted for serum albumin levels by calculating the free thiol groups/albumin ratio. This adjustment was performed since albumin is the predominant blood protein and therefore largely determines the total quantity of potentially detectable free thiol groups [17].

### 2.4. Statistical Analysis

Demographic and clinical characteristics of the study population were presented as means ± standard deviations (SD), proportions *n* with corresponding percentages (%) or medians (interquartile range (IQR)) in case of skewed distributions. Normality testing was performed using histograms, normal probability plots (Q–Q plots) and the D’Agostino and Pearson’s omnibus K^2^ normality test [36]. Comparisons between groups for continuous variables were performed using independent sample *t*-tests, Mann–Whitney *U*-tests, one-way analysis of variance (ANOVA) or the Kruskal-Wallis test, while for nominal variables chi-square tests or Fisher’s exact tests were performed, as appropriate. IBD patients were subdivided into two groups of below- and above-average serum free thiol concentrations and compared for demographic, clinical and biochemical parameters. Univariable and multivariable linear regression analyses were performed to identify variables that were independently associated with serum free thiol concentrations. Multivariable linear regression was executed using backward selection (*P*_out_ > 0.05), with inclusion of all significantly associated variables from univariable analysis. Non-normally distributed continuous variables were logarithmically transformed before entry into linear regression analysis. Independent associations were visualized in scatter plots showing Pearson’s product-moment correlation coefficient. To evaluate discriminative capacity of albumin-adjusted serum free thiols regarding endoscopic disease activity, receiver operating characteristics (ROC) analyses were performed with the calculated area under the curve (AUC) as overall measure of fit. ROC curves and AUC values were calculated using the nonparametric, tie-corrected trapezoidal approximation method. Univariable logistic regression analysis was used to identify potentially confounding factors, which were included as covariate in a multivariable logistic regression model to adjust for these factors. The final predictive model of albumin-adjusted serum free thiols with covariate adjustment (derived from multivariable logistic regression analysis) was internally validated using *k*-fold cross-validation (*k* = 10). To do this, the dataset was randomly partitioned into *k* approximately equally sized parts. Each part was then left out (10% of cases) while the model was fitted to the remaining *k* – 1 parts (90% of cases, ‘training set’) and predictions were obtained for the left-out *k*th part (‘test set’). This procedure was repeated 10 times, so each part acted as test set. Finally, AUCs from each fold were averaged and bootstrapped to achieve statistical inference, resulting in a cross-validated AUC with a corresponding 95% confidence interval (CI). Where appropriate, corrections for multiple comparisons were applied using the Bonferroni correction. Data analysis was performed using SPSS Statistics 25.0 software package (SPSS Inc., Chicago, IL, USA) and Stata 15.1 (Stata Corp LLC, College Station, TX, USA) using the commands ‘roctab’, ‘rocreg’ and the CVAUROC module (github link: https://github.com/migariane/cvAUROC) [37]. Data were visualized using GraphPad Prism 5.0 (GraphPad software, San Diego, CA, USA). Two-tailed *P*-values ≤ 0.05 were considered statistically significant.

### 2.5. Ethical Considerations

Serum samples of IBD patients were obtained after they provided written informed consent. This study was approved by the Institutional Review Board (IRB) of the University Medical Center Groningen (UMC Groningen) (in Dutch: ‘Medisch Ethische Toetsingscommissie’, METc) (IRB no. 2018/703). In addition, serum samples of 50 healthy, non-IBD controls were retrieved from a UMCG biobank containing pre-donation samples of living kidney donors (PSI-UMCG (IRB no. 2008/279)). The study was performed in accordance with the principles of the Declaration of Helsinki (2013).

## 3. Results

### 3.1. Cohort Demographic and Clinical Characteristics

The study cohort consisted of 78 IBD patients (47 UC patients and 31 CD patients) and 50 healthy controls (HC). Study groups were matched for gender (IBD: 46.2% females vs. HC: 46.0% females, *P* = 0.99). Age was significantly different between patients with IBD and HC (mean ± SD: 43.3 ± 8.5 vs. 50.7 ± 8.5 years, respectively, *P* < 0.01) and was adjusted for in all analyses when HC were involved. Most IBD patients had clinically active disease (76.7% with HBI or SCCAI score > 5). All shared characteristics between patients with IBD and HC are presented in Table 1.

### 3.2. Serum Levels of Free Thiols are Markedly Decreased in IBD

**Figure 1:**
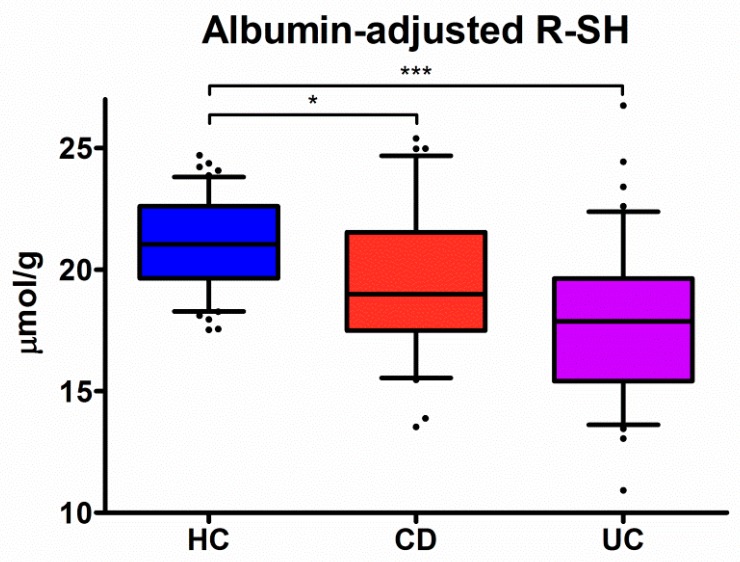
Albumin-adjusted serum free thiols (R-SH) (µmol/g) are highly significantly reduced in both Crohn’s disease (CD) and ulcerative colitis (UC) patients as compared to healthy controls (* *P* < 0.05 and *** *P* < 0.001, respectively).

Serum levels of albumin-adjusted free thiols were significantly lower in both CD (19.4 ± 3.1 µmol/g, *P* < 0.05) and UC (17.8 ± 3.4 µmol/g, *P* < 0.001) patients, as compared to HC (21.1 ± 1.9 µmol/g) (Figure 1). In addition, serum albumin-adjusted free thiol levels were significantly reduced in UC as compared to CD (*P* < 0.05). However, this was not significant after correction for multiple testing (*P* = 0.05). In all groups, albumin-adjusted serum free thiol levels were normally distributed (CD, *P* = 0.37; UC, *P* = 0.43; HC, *P* = 0.14) (Figure S1).

### 3.3. Associations between Serum Albumin-Adjusted Free Thiols and IBD Parameters

Disease parameters of IBD patients are summarized in Table 2 with a comparison between patients with below-average albumin-adjusted serum free thiols (<18.5 μmol/g) (*n* = 40) and above-average albumin-adjusted serum free thiols (>18.5 μmol/g) (*n* = 38). IBD patients with below-average albumin-adjusted serum free thiols had significantly higher WBC (median (IQR) 8.7 (6.5–12.5) vs. 6.5 (5.3–9.2) × 10^9^/L, *P* < 0.01), platelets (339 (283–402) vs. 290 (236–331) × 10^9^/L, *P* < 0.05) and fecal calprotectin levels (1465 (929–2420) vs. 600 (255–1220) µg/g, *P* < 0.01), whereas disease duration was shorter (6.0 (2.0–11.0) vs. 9.0 (3.8–13.0) years, *P* < 0.05) and eGFR was significantly lower (mean ± SD 95.8 ± 22.9 vs. 109.0 ± 28.4 mL/min/1.73 m^2^, *P* < 0.05). No significant differences in albumin-adjusted serum free thiol concentrations were observed for different disease localization according to the Montreal classification (CD, *P* = 0.15; UC, *P* = 0.46) (Figure S2).

Using univariable and multivariable linear regression analyses, age (*r* = −0.49, *P* < 0.01), disease duration (*r* = 0.39, *P* < 0.05), platelets (*r* = −0.29, *P* < 0.01) and fecal calprotectin levels (*r* = −0.32, *P* < 0.05, *n* = 36) were independently associated with albumin-adjusted serum free thiols in the IBD cohort (Table 3 and Figure S3). In the CD cohort, platelets (*r* = −0.51, *P* < 0.05) and disease duration (*r* = 0.39, *P* < 0.05) were independently associated, whereas in the UC cohort, the white blood cell count (WBC) (*r* = −0.41, *P* < 0.05) and Simple Clinical Colitis Activity Index (SCCAI) (*r* = −0.34, *P* < 0.05) were independently associated with albumin-adjusted serum free thiols (Tables S1 and S2).

### 3.4. Serum Free Thiols Strongly Correlate with Endoscopic Disease Activity

**Figure 2:**
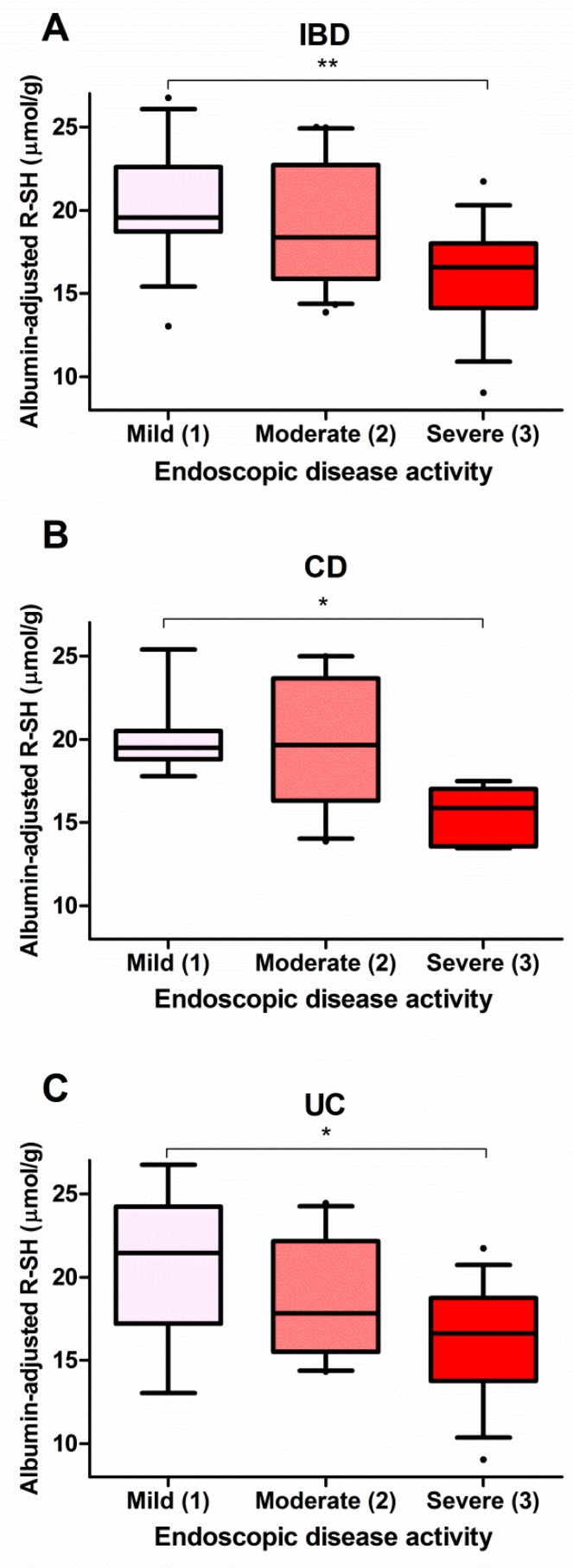
Albumin-adjusted serum free thiols are significantly decreased in (**A**) IBD patients, (**B**) CD patients and (**C**) UC patients with severe endoscopic disease activity as compared to mild disease activity (** *P* < 0.01; * *P* < 0.05).

In a subset of patients (*n* = 54, 69.2%), an endoscopy procedure was performed close to the day that serum samples were collected for routine biochemistry and free thiol analysis. Albumin-adjusted serum free thiols were significantly associated with endoscopic disease activity (Figure 2). IBD patients with severe endoscopic disease activity had significantly lower levels of albumin-adjusted serum free thiols compared to patients with mild disease activity (16.2 ± 3.1 µmol/g vs. 20.4 ± 3.4 µmol/g, *P* < 0.01) (Figure 2A). Stratified by IBD subtype, both CD and UC patients with severe disease activity exhibited significantly reduced levels of albumin-adjusted serum free thiols compared to patients with mild disease activity (CD, 16.6 ± 0.8 µmol/g vs. 20.0 ± 2.3 µmol/g, *P* < 0.05; UC, 16.2 ± 3.4 µmol/g vs. 20.8 ± 4.7 µmol/g, *P* < 0.05) (Figure 2B,C).

### 3.5. Discriminative Accuracy of Serum Free Thiols regarding Endoscopic Disease Activity

**Figure 3:**
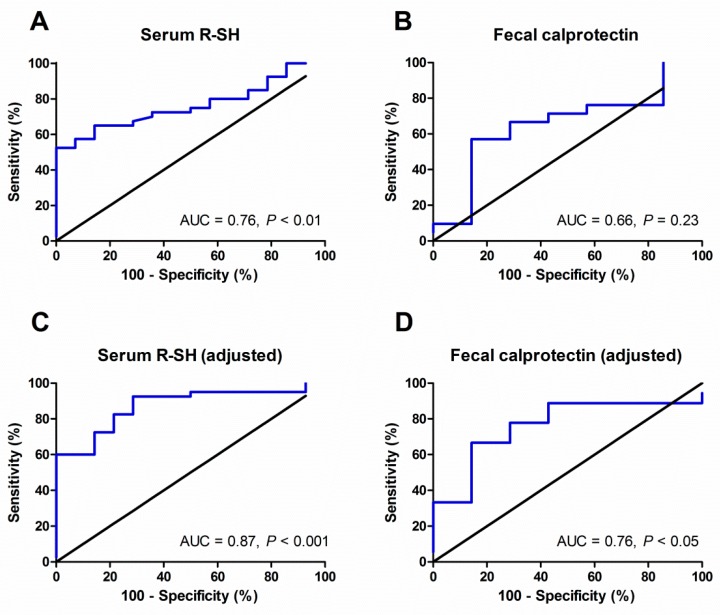
Albumin-adjusted serum free thiol (R-SH) levels (**A**) discriminate better between mild (1) and moderate-to-severe (2,3) endoscopic disease activity as compared to fecal calprotectin levels (**B**), even after adjustment for potentially confounding factors (**C–D**).

As a next step, albumin-adjusted serum free thiols were evaluated in terms of their discriminative accuracy to differentiate mild disease activity (SES-CD or Mayo category 1) from moderate-to-severe endoscopic disease activity (SES-CD or Mayo categories 2 and 3) (Figure 3). In the IBD cohort, serum levels of albumin-adjusted free thiols significantly discriminated patients with mild disease activity from patients with moderate-to-severe disease activity (area under the curve (AUC) = 0.76, 95% confidence interval (CI) 0.64–0.89, *P* < 0.01, Figure 3A and Table S3). For comparison, the AUC for fecal calprotectin levels was 0.66 (95% CI 0.42–0.89, *P* = 0.23, *n* = 28, Figure 3B and Table S3). Fecal calprotectin levels closest to the date of endoscopy were incorporated into this analysis (*n* = 28, median (IQR) time interval: 30 (21–50) days, all within 3 months). After adjustment for potentially confounding factors derived from univariable logistic regression analysis (Table S4), serum free thiols remained superior to fecal calprotectin levels in discriminating between mild and moderate-to-severe endoscopic disease activity (AUC 0.87, 95% CI 0.78–0.97, *P* < 0.001 vs. AUC 0.76, 95% CI 0.56–0.96, *P* < 0.05, respectively) (Figure 3C,D). This final adjusted model containing serum free thiols maintained its discriminative ability after *k*-fold cross-validation (*k* = 10), which resulted in an AUC of 0.89 (95% CI 0.68–0.93, *P* < 0.001) (Figure S4 and Table S5).

## 4. Discussion

We demonstrate that albumin-adjusted serum free thiols are significantly correlated to endoscopic disease activity in IBD. Also, serum albumin-adjusted free thiols are superior in terms of their ability to discriminate between mild and moderate-to-severe disease activity as compared to FC levels (AUC = 0.87, *P* < 0.001 vs. AUC = 0.76, *P* < 0.05). In the final adjusted model, albumin-adjusted serum free thiols very accurately discriminated between mild and moderate-to-severe disease activity, confirmed by an AUC of 0.89 after cross-validation. Furthermore, albumin-adjusted serum free thiol concentrations were significantly lower in IBD as compared to healthy individuals, with lower values in patients with UC as compared to those with CD. Finally, in the IBD cohort, serum concentrations of albumin-adjusted free thiols were significantly inversely associated with age, platelet counts and fecal calprotectin (FC) levels, and positively associated with duration of disease.

Our study is the first to explore the potential use of serum free thiols as a surrogate measure of systemic oxidative stress to assess disease activity in IBD. Earlier studies have assessed a variety of other oxidative stress biomarkers in IBD and related them to disease activity. For instance, it has repeatedly been demonstrated that IBD patients show a lower total blood antioxidant status, as reflected by decreased total antioxidant capacity (TAC) and low levels of antioxidants such as β-carotene, glutathione peroxidase (GPx), superoxide dismutase (SOD) and catalase (CAT) [38,39,40,41]. However, in these studies, correlations with disease activity remain inconsistent. Most notably, nearly all of these studies established their correlations with disease activity based on clinical symptom scores. Similarly, such conflicting results even exist within our own study, since we observed an independent significant association between serum free thiols and the SCCAI score for UC, whereas no significant correlation was found with the HBI score for CD. However, there are hardly any studies evaluating oxidative stress biomarkers that focused on relationships with validated endoscopy scores, used to define the degree of endoscopic disease activity [42,43]. In the case of UC, there are few studies in which a significant correlation was detected between the endoscopic activity index (EAI) and total oxidant status (TOS) levels, as measured by a spectrophotometric assay [42]. Another study investigated associations between the Mayo endoscopic subscore and serum levels of the antioxidant enzymes GPx, SOD-1 and CAT, though these associations were all non-significant [43]. In contrast, we observed a clear correlation between serum free thiols and endoscopic disease activity scores, both in the total IBD cohort and for CD and UC patients separately. Perhaps this observation may be explained by the fact that the measurement of serum free thiol concentrations provides a more accurate indication of systemic redox status as compared to individual oxidant or antioxidant substances and derived products [16].

Results from the present study further emphasize the potential significance of serum free thiols as an integrative marker of human systemic redox status. Serum free thiol groups (R-SH, serum sulfhydryl status) are representative of an intricate and dynamic extracellular redox regulation system with potent antioxidant activity, since they act as a major scavenger of circulating reactive species [16]. Extracellular thiols are largely embedded in proteins, whereas a smaller fraction of the total thiol pool is represented by low molecular weight (LMW) thiols, such as cysteine, glutathione (GSH) and homocysteine, which exist in both reduced and oxidized forms [44]. However, LMW thiols predominantly control intracellular redox status and are of lesser importance to redox regulation within the extracellular compartment [17]. Blood proteins contain the largest amount of thiols. In this respect, serum albumin is quantitatively the most important (based on its single free cysteine residue, Cys^34^), not only because of its abundance, but also given its transporting capacity of LMW thiols [17]. In the present study, we only measured serum free thiol concentrations and adjusted these to serum albumin levels, which can be regarded as an indirect approach to more precisely reflect the total serum thiol pool [45].

In this study, we found independent associations between serum free thiols and age, duration of disease, platelets and fecal calprotectin levels. The inverse correlation between age and serum free thiols corroborates the findings of previous studies [18,23]. Serum free thiol concentrations decrease with age, which is putatively caused by a combination of compromised antioxidant availability, an age-related increase in ROS production and a decreased ratio of reduced vs. oxidized forms of serum albumin [23]. Counterintuitively, we observed a positive association between serum free thiols and duration of disease. Contradictory to this finding, there is considerable evidence suggesting that longer-term exposure to oxidative stress, with continuous ROS generation and deteriorating antioxidant defense, further aggravates ROS-mediated tissue damage and concomitant inflammatory activity [26,46,47]. In fact, oxidative stress is believed to be a key mechanism in disease progression of IBD. Nevertheless, it should be taken into account that most IBD patients follow a highly unpredictable, chronic intermittent disease course, from which a dynamic association may arise between disease duration and inflammatory activity or oxidative stress [48]. Indeed, in our study, patients with relatively longer disease duration had more often less severe disease activity compared to patients with shorter IBD history, though this difference was not statistically significant. Lastly, we detected an independent significant association between platelet counts and serum free thiol concentrations. It is well recognized that high platelet counts (thrombocytosis) coincide with inflammatory activity and oxidative stress in IBD [49]. For instance, platelets have been shown to be of predictive value regarding endoscopic disease activity in IBD [50]. 

Intriguingly, our data demonstrate that FC levels were significantly negatively correlated to albumin-adjusted serum free thiols. The observed significant correlation is consistent with a recent study in which significant correlations were observed between FC levels and the serum antioxidant enzymes GPx and CAT [51]. These authors hypothesized that these correlations, based on another study on polymorphonuclear neutrophils, could originate from the fact that FC has pro-oxidant properties by boosting the formation of ROS [52]. FC is a protein dimer complex (S100A8/S100A9) representing approximately 60% of cytosolic proteins of neutrophilic granulocytes and is released upon the process of intestinal inflammation [53]. Therefore, increased neutrophilic inflammatory disease activity, as represented by elevated FC levels, accompanied by increased oxidative stress, may likely be mirrored by a decrease in the total pool of serum free thiols. Subsequently, the increased level of oxidative stress may induce a compensatory production of antioxidant enzymes to counteract ROS activity and restore thiol homeostasis [51].

In this study, we showed that serum free thiols were able to accurately discriminate between mild and moderate-to-severe endoscopic disease activity in our cohort of IBD patients. Although this promising result should be further validated in larger, prospective validation cohorts, it may be particularly relevant as discriminating between different degrees of active disease may help in guiding therapeutic decisions and adjusting medical therapy [54]. In addition, endoscopic assessment of disease activity (commonly referred to as ‘mucosal healing’) is emerging as an important therapeutic endpoint in clinical trials [55]. As future assessment of endoscopic disease activity may be found in the measurement of serum biomarkers, this study is aimed to serve as a stepping stone for future studies that could evaluate the potential of serum free thiols as biomarkers for (endoscopic) disease activity in IBD.

Strengths of our study include the extensive characterization of the patient cohort and the control group, which allowed us to directly compare serum free thiol concentrations between IBD and healthy individuals. Additionally, our study was fueled by sufficient study power to be able to reliably establish differences between groups and to properly adjust for potential confounding factors. However, our study has also limitations. The study was of observational, cross-sectional design with a relatively small cohort of IBD patients in which the primary outcome (endoscopic disease activity) was only available from a subset (*n* = 54) of patients. Most importantly, no IBD patients with disease in remission were included, which could have enabled us to assess the discriminative ability of serum free thiols between quiescent and active disease and to establish clinically relevant cut-off values. In line, we argued that our study was not appropriately designed to produce clinically useful cut-off values for mild vs. moderate-to-severe disease activity as this would require a prospective diagnostic study with serial assessments and larger sample sizes. Instead, we selected patients with at least some degree of disease activity, aiming to examine serum free thiols as potential biomarker for disease activity. Finally, time intervals between serum sampling and the endoscopy procedure varied among patients, leading to a certain degree of temporal heterogeneity that could potentially have biased our results, or may have resulted in an underestimation of the currently established correlation with disease activity.

## 5. Conclusions

In conclusion, we show that albumin-adjusted serum free thiols are significantly reduced in IBD as compared to healthy individuals and are in line with the degree of endoscopically proven disease activity. Furthermore, we show that serum free thiol levels significantly negatively correlate to fecal calprotectin levels and may aid in differentiating mild from moderate-to-severely active IBD as assessed by endoscopy. Future studies are warranted to further externally validate serum free thiol levels as potential biomarker for IBD disease activity in larger, prospective patient cohorts and assess their value in relation to disease course and therapeutic interventions.

## Figures and Tables

**Table 1:** Baseline characteristics of IBD patients and healthy controls (HC).

Variables	HC	IBD	*P*-Value
	*n* = 50	*n* = 78	
Serum free thiols per gram of albumin (µmol/g)	21.1 ± 1.9	18.5 ± 3.4	**<0.001**
Age (years)	50.7 ± 8.5	43.3 ± 8.5	**<0.01**
Female, *n* (%)	23 (46.0)	36 (46.2)	0.99
BMI (kg/m^2^)	26.9 ± 3.1	24.8 ± 4.9	**<0.01**
Current smoking, *n* (%)	13 (26.0)	13 (18.8)	0.35
**Laboratory Measurements**			
Hemoglobin (g/dL)	14.8 ± 1.3	12.3 ± 1.8	**<0.001**
CRP (mg/L) *	1.0 (0.6–1.5)	4.1 (1.2–10.3)	**<0.001**
WBC (×10^9^/L) *	6.7 (5.4–7.8)	7.6 (6.0–10.3)	**<0.01**
Platelets (×10^9^/L) *	244 (207–296)	308 (259–376)	**<0.001**
Albumin (g/L)	45.6 ± 2.3	40.1 ± 3.9	**<0.001**
eGFR (mL/min * 1.73m^2^)	87.7 ± 14.5	102.4 ± 26.4	**<0.01**
Creatinine (µmol/L)	81.5 ± 15.9	73.1 ± 21.1	**<0.05**

Data are presented as mean ± SD or proportions (*n*, %). * Skewed data are presented as median (interquartile range). *P*-values were two-tailed and calculated using independent sample *t*-tests or Mann–Whitney *U*-tests, as appropriate. Abbreviations: IBD, inflammatory bowel disease; CD, Crohn’s disease; HC, healthy controls; BMI, body mass index; CRP, C-reactive protein; WBC, white blood cell count; eGFR, estimated glomerular filtration rate. Bold *P*-values indicate statistical significance.

**Table 2:** Baseline demographic, clinical and IBD-specific characteristics compared between above- and below-average albumin-adjusted serum free thiol concentrations (average: 18.5 ± 3.4 µmol/g).

Variables	Total IBD Cohort	Below-Average Thiols	Above-Average Thiols	*P*-value
	*n* = 78	*n* = 40	*n* = 38	
Serum free thiols per gram of albumin (µmol/g)	18.5 ± 3.4	15.9 ± 2.1	21.1 ± 2.2	**<0.001**
Age (years)	43.3 ± 8.5	44.4 ± 16.7	42.1 ± 13.8	0.51
Female, *n* (%)	36 (46.2)	17 (42.5)	19 (50.0)	0.51
BMI (kg/m^2^)	24.8 ± 4.9	24.4 ± 4.9	25.2 ± 4.9	0.49
Current smoking, *n* (%)	13 (18.8)	7 (21.1)	6 (16.7)	0.63
Prior surgery, *n* (%)	21 (26.9)	7 (17.5)	14 (36.8)	0.06
Disease duration (years)^*^	7.0 (3.0–12.3)	6.0 (2.0–11.0)	9.0 (3.8–13.0)	**<0.05**
Prior anti-TNF, *n* (%)	67 (85.6)	32 (80.0)	35 (92.1)	0.28
HBI/SCCAI^*^	6 (5–10)	6 (4.8–10.0)	6.5 (4.8–10.3)	0.93
**Disease Location (CD), *n* (%)**				0.05
	*n* = 31	*n* = 12	*n* = 19	
L1 (ileal)	6 (19.4)	-	6 (31.6)	
L2 (colonic)	4 (12.9)	3 (25.0)	1 (5.3)	
L3 (ileocolonic)	21 (67.7)	9 (75.0)	12 (63.2)	
**Disease Extent (UC), *n* (%)**				0.54
	*n* = 47	*n* = 28	*n* = 19	
E1 (proctitis)	-	-	-	
E2 (left-sided colitis)	15 (31.9)	10 (35.7)	5 (26.3)	
E3 (pancolitis)	32 (68.1)	18 (64.3)	14 (73.7)	
**Age at Diagnosis (Montreal A)**				0.32
A1 (< 16 years)	10 (12.8)	5 (12.5)	5 (13.2)	
A2 (17 – 40 years)	50 (64.1)	23 (57.5)	27 (71.1)	
A3 (> 40 years)	18 (23.1)	12 (30.0)	6 (15.8)	
**Medication, *n* (%)**				0.28
None	34 (43.6)	20 (50.0)	14 (36.8)	
Thiopurines	19 (24.4)	7 (17.5)	12 (31.6)	
Mesalamine	18 (23.1)	8 (20.0)	10 (26.3)	
Combination	7 (9.0)	5 (12.5)	2 (5.3)	
**Laboratory Measurements**				
Hemoglobin (g/dL)	12.3 ± 1.8	12.1 ± 1.6	12.4 ± 1.9	0.32
CRP (mg/L)^*^	4.1 (1.2–10.3)	4.1 (1.0–12.5)	3.9 (1.2–9.2)	0.77
ESR (mm/h)^*^	17.0 (6.0–41.3)	22.5 (7.0–40.1)	13.5 (4.0–44.3)	0.42
WBC (×10^9^/L)^*^	7.6 (6.0–10.3)	8.7 (6.5–12.5)	6.5 (5.3–9.2)	**<0.01**
Platelets (×10^9^/L)^*^	308 (259–376)	339 (283–402)	290 (236–331)	**<0.05**
Albumin (g/L)	40.1 ± 3.9	40.6 ± 3.4	39.6 ± 4.3	0.270
eGFR (mL/min/1.73 m^2^)	102.4 ± 26.4	95.8 ± 22.9	109.0 ± 28.4	**<0.05**
Creatinine (µmol/L)	73.1 ± 21.1	77.1 ± 24.1	69.0 ± 16.7	0.091
Fecal calprotectin (µg/g)^*,†^	1220 (610–1885)	1465 (929–2420)	600 (255–1220)	**<0.01**

Data are presented as mean ± SD or proportions (*n*, %). *Skewed data are presented as median (interquartile range). ^†^Fecal calprotectin levels were only measured in a subset of patients (*n* = 36). *P*-values were two-tailed and calculated using independent sample *t*-tests or Mann–Whitney *U*-tests, as appropriate. *P*-values < 0.05 were considered statistically significant (indicated in bold). Abbreviations: CD, Crohn’s disease; UC, ulcerative colitis; BMI, body mass index; HBI, Harvey Bradshaw index; SCCAI, simple clinical colitis activity index; TNF, tumor necrosis factor; CRP, C-reactive protein; ESR, erythrocyte sedimentation rate; WBC, white blood cell count; eGFR, estimated glomerular filtration rate.

**Table 3:** Univariable and multivariable linear regression analyses of albumin-adjusted serum free thiols in IBD with clinical and biochemical parameters.

Serum Free Thiols/Gram of Albumin	Univariable Analysis		Multivariable Analysis	
Variables	B Coefficient ^#^	*P*-Value	B Coefficient ^#^	*P*-Value
Age	−0.237	**0.04** ^†^	−0.500	**<0.01** ^†^
Female sex	0.093	0.42		
Current smoker	−0.054	0.66		
BMI	−0.042	0.73		
Disease duration *	0.288	**<0.05 ^†^**	0.364	**<0.05** ^†^
Prior anti-TNF	0.180	0.11		
HBI/SCCAI *	−0.177	0.19		
**Medication**				
Thiopurines	0.070	0.55		
Mesalamine	0.119	0.30		
Combination	−0.105	0.36		
**Laboratory Measurements**				
Hemoglobin	0.102	0.37		
CRP *	−0.007	0.95		
ESR *	−0.128	0.27		
WBC *	−0.338	**<0.01 ^†^**		
Platelets *	−0.223	**0.05 ^†^**	−0.278	**<0.05** ^†^
Albumin	−0.127	0.27		
eGFR	0.370	**<0.01 ^†^**		
Creatinine	−0.235	**0.04 ^†^**		
Fecal calprotectin *	−0.422	**0.01 ^†^**	−0.345	**<0.05** ^†^

* Skewed data have been logarithmically transformed before entry into analyses. ^#^ Standardized beta (β) coefficient. ^†^ *P*-values < 0.05 were considered statistically significant (indicated in bold). Abbreviations: BMI, body mass index; HBI, Harvey Bradshaw index; SCCAI, simple clinical colitis activity index; TNF, tumor necrosis factor; CRP, C-reactive protein; ESR, erythrocyte sedimentation rate; WBC, white blood cell count; eGFR, estimated glomerular filtration rate.

## Supplementary Materials

The following are available online at https://www.mdpi.com/2076-3921/8/9/351/s1, Figure S1: Albumin-adjusted serum free thiols (R-SH) are normally distributed among healthy controls (**A**) and IBD patients (**B**). Figure S2: Albumin-adjusted serum free thiol concentrations (µmol/g) do not significantly differ among different disease localizations in both Crohn’s disease (ileal [L1], colonic [L2] or ileocolonic [L3] disease) and ulcerative colitis (left-sided colitis [E2] or pancolitis [E3]). Figure S3: Age, disease duration, platelets and fecal calprotectin levels are independently associated with albumin-adjusted serum free thiols (correlations are shown from univariable analysis). Figure S4: Albumin-adjusted serum free thiol (R-SH) levels accurately discriminate between mild (1) and moderate-to-severe (2-3) endoscopic disease activity (AUC = 0.89, blue curve) after *k*-fold cross-validation (*k* = 10) (dashed curves). Table S1: Univariable and multivariable linear regression analyses of albumin-adjusted serum free thiols (R-SH) in Crohn’s disease (CD) with clinical and biochemical parameters. Table S2: Univariable and multivariable linear regression analyses of albumin-adjusted serum free thiols (R-SH) in ulcerative colitis (UC) with clinical and biochemical parameters. Table S3: ROC curve coordinates for both serum free thiols (R-SH) and fecal calprotectin levels, showing all x-axis coordinates (step values of the false positive rate, FPR) with corresponding sensitivity ranges (%). Table S4: Univariable logistic regression analysis with demographic and clinical factors of IBD patients with regards to endoscopic (mild vs. moderate-to-severe) disease activity prior to serum sample analysis. Table S5: Results from *k*-fold cross-validation (*k* = 10) of the final adjusted model containing serum free thiols, resulting in a cross-validated area under the curve (AUC) of 0.89 (95% CI: 0.63–0.93).
