# Supplementary material for: Serum Free Thiols Are Superior to Fecal Calprotectin in Reflecting Endoscopic Disease Activity in Inflammatory Bowel Disease
Source: Antioxidants (Basel). 2019 Sep 1;8(9):351. doi: 10.3390/antiox8090351 (PMC6769968; doi:10.3390/antiox8090351)
Supplement: Supplementary file 1 [file antioxidants-08-00351-s001.zip › Table S5.docx]

**Table S4**. Results from *k*-fold cross-validation (*k* = 10) of the final adjusted model containing serum free thiols, resulting in a cross-validated area under the curve (AUC) of 0.89 (95% CI: 0.63 – 0.93).

| Randomization number | Validation cases | AUC |
| --- | --- | --- |
| 1 | *n* = 6 | 1.00 |
| 2 | *n* = 5 | 1.00 |
| 3 | *n* = 6 | 0.63 |
| 4 | *n* = 5 | 1.00 |
| 5 | *n* = 5 | 1.00 |
| 6 | *n* = 6 | 1.00 |
| 7 | *n* = 5 | 1.00 |
| 8 | *n* = 6 | 0.80 |
| 9 | *n* = 5 | 1.00 |
| 10 | *n* = 5 | 0.50 |
| Total | *n* = 54 | 0.89 (mean) |

Abbreviations: AUC, area under the curve.
