# Supplementary material for: Serum Free Thiols Are Superior to Fecal Calprotectin in Reflecting Endoscopic Disease Activity in Inflammatory Bowel Disease
Source: Antioxidants (Basel). 2019 Sep 1;8(9):351. doi: 10.3390/antiox8090351 (PMC6769968; doi:10.3390/antiox8090351)
Supplement: Supplementary file 1 [file antioxidants-08-00351-s001.zip › Table S1.docx]

**Table S1**. Univariable and multivariable linear regression analyses of albumin-adjusted serum R-SH in Crohn’s disease (CD) with clinical and biochemical parameters.

| Serum R-SH / gram of albumin | Univariable analysis | | Multivariable analysis | |
| --- | --- | --- | --- | --- |
| Variables | **B coefficient^#^** | ***P*-value** | **B coefficient^#^** | ***P*-value** |
| Age | -0.081 | 0.67 |  |  |
| Female sex | -0.106 | 0.57 |  |  |
| Current smoker | -0.083 | 0.66 |  |  |
| BMI | 0.034 | 0.86 |  |  |
| Disease duration^*^ | 0.365 | **0.04^†^** | 0.393 | **0.02^†^** |
| Prior surgery | 0.229 | 0.22 |  |  |
| Prior anti-TNF | 0.280 | 0.13 |  |  |
| HBI* | -0.132 | 0.51 |  |  |
| Co-medication |  |  |  |  |
| Thiopurines | -0.027 | 0.88 |  |  |
| Mesalamine | 0.338 | 0.06 |  |  |
| Laboratory measurements |  |  |  |  |
| Haemoglobin | 0.131 | 0.48 |  |  |
| CRP^*^ | 0.009 | 0.96 |  |  |
| ESR^*^ | -0.112 | 0.55 |  |  |
| WBC^*^ | -0.316 | 0.08 |  |  |
| Platelets^*^ | -0.378 | **0.04^†^** | -0.513 | **0.02^†^** |
| Albumin | -0.276 | 0.13 |  |  |
| eGFR | 0.140 | 0.45 |  |  |
| Creatinine | 0.088 | 0.64 |  |  |
| Fecal calprotectin^*^ | -0.524 | **0.04^†^** |  |  |

*Skewed data have been logarithmically transformed before entry into analyses. ^#^Standardized beta (β) coefficient. ^†^*P*-values < 0.05 were considered statistically significant. Abbreviations: R-SH, free thiols; BMI, body mass index; HBI, Harvey Bradshaw Index; TNF, tumor necrosis factor; CRP, C-reactive protein; ESR, erythrocyte sedimentation rate; WBC, white blood cell count; eGFR, estimated glomerular filtration rate.
