# Supplementary material for: Serum Free Thiols Are Superior to Fecal Calprotectin in Reflecting Endoscopic Disease Activity in Inflammatory Bowel Disease
Source: Antioxidants (Basel). 2019 Sep 1;8(9):351. doi: 10.3390/antiox8090351 (PMC6769968; doi:10.3390/antiox8090351)
Supplement: Supplementary file 1 [file antioxidants-08-00351-s001.zip › Table S2.docx]

**Table S2**. Univariable and multivariable linear regression analyses of albumin-adjusted serum R-SH in ulcerative colitis (UC) with clinical and biochemical parameters.

| Serum R-SH / gram of albumin | Univariable analysis | | Multivariable analysis | |
| --- | --- | --- | --- | --- |
| Variables | **B coefficient^#^** | ***P*-value** | **B coefficient^#^** | ***P*-value** |
| Age | -0.392 | **< 0.01^†^** |  |  |
| Female sex | 0.061 | 0.68 |  |  |
| Current smoker | -0.154 | 0.35 |  |  |
| BMI | -0.029 | 0.86 |  |  |
| Disease duration^*^ | 0.146 | 0.33 |  |  |
| Prior surgery | 0.135 | 0.40 |  |  |
| Prior anti-TNF | -0.059 | 0.70 |  |  |
| SCCAI^*^ | -0.385 | **0.04^†^** | -0.336 | **< 0.05^†^** |
| Co-medication |  |  |  |  |
| Thiopurines | 0.009 | 0.95 |  |  |
| Mesalamine | 0.178 | 0.23 |  |  |
| Combination | -0.061 | 0.68 |  |  |
| Laboratory measurements |  |  |  |  |
| Haemoglobin | 0.112 | 0.45 |  |  |
| CRP^*^ | -0.118 | 0.43 |  |  |
| ESR^*^ | -0.254 | 0.09 |  |  |
| WBC^*^ | -0.374 | **0.01^†^** | -0.410 | **0.02^†^** |
| Platelets^*^ | -0.202 | 0.17 |  |  |
| Albumin | 0.017 | 0.91 |  |  |
| eGFR | 0.500 | **< 0.001^†^** |  |  |
| Creatinine | -0.288 | **0.05^†^** |  |  |
| Fecal calprotectin^*^ | -0.320 | 0.16 |  |  |

*Skewed data have been logarithmically transformed before entry into analyses. ^#^Standardized beta (β) coefficient. ^†^*P*-values < 0.05 were considered statistically significant. Abbreviations: R-SH, free thiols; BMI, body mass index; SCCAI, Simple Clinical Colitis Activity Index; TNF, tumor necrosis factor; CRP, C-reactive protein; ESR, erythrocyte sedimentation rate; WBC, white blood cell count; eGFR, estimated glomerular filtration rate.
