# Supplementary material for: Serum Free Thiols Are Superior to Fecal Calprotectin in Reflecting Endoscopic Disease Activity in Inflammatory Bowel Disease
Source: Antioxidants (Basel). 2019 Sep 1;8(9):351. doi: 10.3390/antiox8090351 (PMC6769968; doi:10.3390/antiox8090351)
Supplement: Supplementary file 1 [file antioxidants-08-00351-s001.zip › Table S3.docx]

**Table S3**. ROC curve coordinates for both serum free thiols and fecal calprotectin levels, showing all x-axis coordinates (step values of the false positive rate, FPR) with corresponding sensitivity ranges (%).

| Serum free thiols (R-SH) (Figure 3A) | | Fecal calprotectin levels (Figure 3B) | |
| --- | --- | --- | --- |
| FPR (%) | Sensitivity range (%) | FPR (%) | Sensitivity range (%) |
| 0.0 | 0.0 – 52.5 | 0.0 | 0.0 – 9.5 |
| 7.1 | 52.5 – 57.5 | 14.3 | 9.5 – 57.1 |
| 14.3 | 57.5 – 65.0 | 28.6 | 57.1 – 66.7 |
| 28.6 | 65.0 – 67.5 | 42.9 | 66.7 – 71.4 |
| 35.7 | 67.-5 – 72.5 | 57.1 | 71.4 – 76.2 |
| 50.0 | 72.5 – 75.0 | 85.7 | 76.2 – 100.0 |
| 57.1 | 75.0 – 80.0 |  |  |
| 71.4 | 80.0 – 85.0 |  |  |
| 78.6 | 85.0 – 92.5 |  |  |
| 85.7 | 92.5 – 100.0 |  |  |

Abbreviations: FPR, false positive rate.
