# Supplementary material for: Serum Free Thiols Are Superior to Fecal Calprotectin in Reflecting Endoscopic Disease Activity in Inflammatory Bowel Disease
Source: Antioxidants (Basel). 2019 Sep 1;8(9):351. doi: 10.3390/antiox8090351 (PMC6769968; doi:10.3390/antiox8090351)
Supplement: Supplementary file 1 [file antioxidants-08-00351-s001.zip › Table S4.docx]

**Table S4**. Univariable logistic regression analysis with demographic and clinical factors of IBD patients with regards to endoscopic (mild vs. moderate-to-severe) disease activity prior to serum sample analysis.

| Variables | Beta coefficient^#^ | OR (95% CI) | *P*-value^†^ |
| --- | --- | --- | --- |
|  |  |  |  |
| Serum free thiols per gram of albumin (µmol/g) | -0.19 | 0.83 (0.69 – 0.99) | **< 0.05** |
| IBD subtype |  |  |  |
| CD | -1.10 | 0.33 (0.10 – 1.14) | 0.08 |
| UC | 1.10 | 3.00 (0.88 – 10.3) | 0.08 |
| Age (years) | 0.02 | 1.02 (0.98 – 1.06) | 0.36 |
| Gender (female) | -1.06 | 0.35 (0.10 – 1.21) | 0.10 |
| BMI (kg/m^2^) | 0.01 | 1.01 (0.89 – 1.14) | 0.88 |
| Disease duration (years)^*^ | -0.06 | 0.94 (0.86 – 1.03) | 0.17 |
| Current smoking, *n* (%) | -0.64 | 0.53 (0.12 – 2.35) | 0.40 |
| Prior surgery, *n* (%) | -2.32 | 0.10 (0.02 – 0.40) | **< 0.01** |
| Concomitant medication use, *n* (%) | -0.46 | 0.63 (0.19 – 2.12) | 0.46 |

*Skewed data have been logarithmically transformed before entry into analyses. ^#^Standardized beta (β) coefficient. ^†^*P*-values < 0.05 were considered statistically significant. Abbreviations: IBD, inflammatory bowel disease; CD, Crohn’s disease; UC, ulcerative colitis; BMI, body mass index; OR, odds ratio; CI, confidence interval.
