# Supplementary figures and images for: Serum Free Thiols Are Superior to Fecal Calprotectin in Reflecting Endoscopic Disease Activity in Inflammatory Bowel Disease
Source: Antioxidants (Basel). 2019 Sep 1;8(9):351. doi: 10.3390/antiox8090351 (PMC6769968; doi:10.3390/antiox8090351)

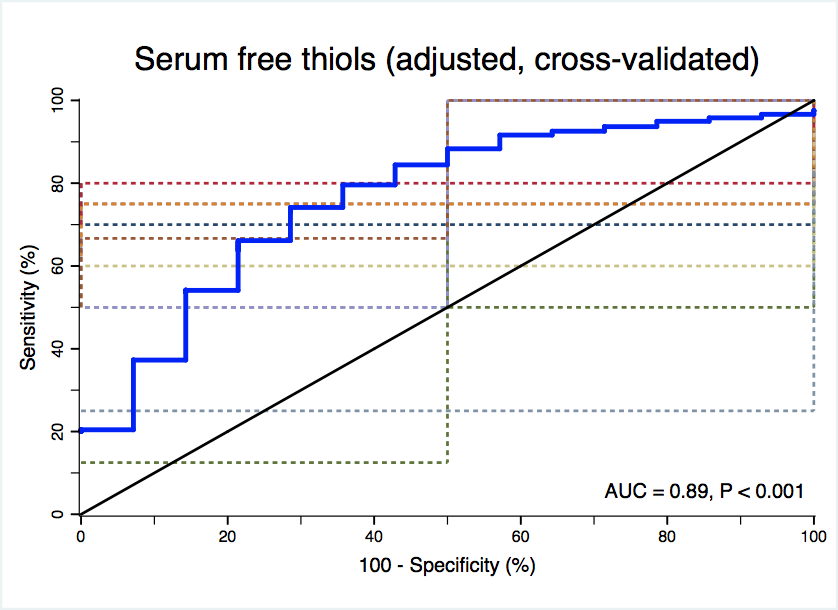

Supplement: Supplementary file 1 [file antioxidants-08-00351-s001.zip › Supplementary Figure S4.tiff]
